# Effects of a Moderately Lower Temperature on the Proliferation and Degranulation of Rat Mast Cells

**DOI:** 10.1155/2016/8439594

**Published:** 2016-04-18

**Authors:** Ruoyu Wang, Xiaoqin Yin, Hui Zhang, Jiwei Wang, Lin Chen, Jingwen Chen, Xiaodong Han, Zou Xiang, Dongmei Li

**Affiliations:** ^1^Immunology and Reproduction Biology Laboratory and State Key Laboratory of Analytical Chemistry for Life Science, Medical School, Nanjing University, Nanjing, Jiangsu 210093, China; ^2^Jiangsu Key Laboratory of Molecular Medicine, Nanjing University, Nanjing, Jiangsu 210093, China; ^3^Department of Microbiology and Immunology, Mucosal Immunobiology and Vaccine Research Center, Institute of Biomedicine, University of Gothenburg, 40530 Gothenburg, Sweden

## Abstract

Mast cells are traditionally considered as key effector cells in IgE-mediated allergic diseases. However, the roles of mast cells have also been implicated in diverse physiological and pathological processes. Mast cells are distributed in various organs and tissues of various species. Some of the organs and tissues, such as testis, skin, and the upper part of the respiratory tract, have a temperature that is lower than the body's core temperature. The purpose of the present study was to investigate the effects of a lower temperature on the proliferation and degranulation of rat mast cells. Here, we demonstrate that cell growth was retarded at 35°C compared to 37°C for both rat peritoneal mast cells (RPMC) and RBL-2H3, a rat mast cell line. Furthermore, RPMC became more susceptible to degranulation at 35°C compared to 37°C. In contrast, degranulation of RBL-2H3 was not as sensitive to temperature change as RPMC. The functionality of mast cells in unique organs with a lower temperature warrants further analysis.

## 1. Introduction

Mast cells are derived from CD34^+^ pluripotent hematopoietic stem cells [1, 2] and they are normal components of connective tissues and are widespread in the organs and tissues of various species [3, 4]. Mast cells are viewed as multifunctional immune cells nowadays despite their well-established role in IgE-mediated allergic pathology [5, 6]. The roles of mast cells are typically elaborated through their release of immune regulatory mediators packed in mast cell granules in the cytoplasm. Upon activation, mast cells can rapidly release granule-associated mediators such as histamine, *β*-hexosaminidase, serotonin, and heparin and synthesize and secrete prostaglandin, leukotriene, various cytokines, and chemokines and biologically active substances that play profound roles in both health and disease [7, 8]. One of the well characterized mechanisms of mast cell activation is through cross-linking of the high affinity IgE receptor, Fc*ε*RI, on the cell surface by IgE and multivalent allergens. However, mast cells can also respond to Fc*ε*RI-independent stimuli that include chemicals such as compound 48/80 (c48/80), IgG immune complexes (via Fc*γ* receptors expressed on mast cells), cytokines, chemokines, and even physical stimuli [9, 10].

In recent decades, studies have implicated mast cell functions in various diseases, such as atherosclerosis [11], pulmonary hypertension [12], infertility [13], autoimmunity [14], bladder pain syndrome (interstitial cystitis) [15], anxiety disorders [16], and obesity and diabetes [17]. Mast cells may play critical roles in these pathologies as a result of their localization in almost all the tissues of the body, with a particularly high density in tissues facing an external environment, such as the skin, the airways, and the gastrointestinal tract [3, 18]. Mast cells also exist in testis, and many studies showed that there is a certain relationship between male infertility and testicular mast cells [13]. Some of these organs and tissues, such as testis, skin, and respiratory tract, have a lower temperature than the core body temperature [19]. For example, the skin temperature is 34°C; the airways have temperatures between 34°C and 37°C; and the testicular temperature is about 35°C. In view of this, we asked the question whether a lower temperature would have any impact on mast cell proliferation and degranulation. In other words, it would be intriguing to investigate whether mast cells can adapt, temperature-wise, to the local environment.

In the current study, we aimed to investigate the effects of a modestly lower temperature (35°C) on the proliferation and degranulation of mast cells isolated from rat peritoneum, that is, rat peritoneal mast cells (RPMC), compared with normal core body temperature of 37°C. We also compared the responsiveness to these two different temperatures between RPMC and the RBL-2H3 cells, a cell line that has been widely used to study the function of mast cells* in vitro*.

## 2. Materials and Methods

### 2.1. Ethics Statement

The animal experiments were performed according to the Guide for the Care and Use of Laboratory Animals (The Ministry of Science and Technology of China, 2006) and all experimental protocols were approved under the animal protocol number SYXK (Su) 2009-0017 by the Animal Care and Use Committee of Nanjing University.

### 2.2. Chemicals and Reagents

RPMI 1640 medium, Minimum Essential Medium (MEM), newborn calf serum, trypsin, collagenase I, and HEPES were purchased from Gibco (Thermo Fisher Scientific, Carlsbad, CA, USA). 4-Nitrophenyl N-acetyl-*β*-D-glucosaminide, compound 48/80 (c48/80), cromolyn sodium salt, penicillin, streptomycin sulfate, and trypsin were purchased from Sigma-Aldrich (St. Louis, MO, USA). Cell Counting Kit-8 was obtained from Dojindo Lab (Kumamoto, Japan). Histamine ELISA kit was purchased from IBL International (Hamburg, Germany). The CytoTox-ONE*™* Homogeneous Membrane Integrity Assay kit was purchased from Promega (Madison, WI, USA). Percoll was purchased from Pharmacia (Stockholm, Sweden). Toluidine blue, glycine, and Triton X-100 were purchased from Sunshine Biotechnology Co. Ltd. (Nanjing, China). FITC-labeled rat mAb to c-Kit was purchased from Abcam (Cambridge, MA, USA). Goat anti-rat IgE polyclonal antibody was purchased from GeneTex Inc. (Irvine, CA, USA). Purified rat IgE was purchased from Life Technologies (Thermo Fisher Scientific, Carlsbad, CA, USA).

### 2.3. Purification of RPMC

RPMC were purified from peritoneal cells of male Sprague-Dawley rats (220–250 g) obtained from Shanghai Super-B&K Laboratory Animal Corp. Ltd. by Percoll density gradient centrifugation as described previously [20]. Briefly, rats were sacrificed and disinfected in alcohol followed by an intraperitoneal injection of 15 mL RPMI1640 and gentle kneading of the abdominal region for 90 s. Next, peritoneal lavage was collected and centrifuged (500 ×g, 10 min, at 4°C). The supernatant was discarded and the cells were collected and resuspended in 1 mL PBS. Cell suspensions were next filtered through a 200-mesh sieve followed by centrifugation at 150 g for 10 min at 4°C, and the pellet was resuspended in PBS. Four milliliters of 90% Percoll was added. After agitation by swirling, 1 mL PBS was dropped in slowly. The mixture was centrifuged (150 ×g) for 6 min. The cells were collected and washed three times with PBS. Approximately 1–5 × 10^5^ cells were obtainable from each rat. Purified RPMC were suspended in RPMI 1640 containing 10% FBS, 100 IU/mL penicillin, and 100 IU/mL streptomycin. Cells were incubated in a humidified atmosphere of 95% air, 5% CO_2_. The purity of mast cells was confirmed by toluidine blue staining and flow cytometry gating on the c-Kit positive population.

### 2.4. RBL-2H3 Culture

RBL-2H3 was purchased from China Center for Type Culture Collection (CCTCC) and seeded into 100 mm culture dishes at a density of approximately 2 × 10^5^ cells per dish in MEM medium containing 10% FBS, 0.11 g/L sodium pyruvate, 1.5 g/L NaHCO_3_, 100 IU/mL penicillin, and 100 IU/mL streptomycin. Cells were incubated in a humidified atmosphere of 95% air, 5% CO_2_. Cells were passaged using trypsin upon reaching 80%–90% confluence and were plated at 2.0 × 10^5^ cells/mL in 24- or 6-well plates for the experiments outlined below.

### 2.5. Cell Proliferation Assay

Cell proliferation was determined by the Cell Counting Kit-8 (CCK-8) method. CCK-8 contains WST-8 which can be deoxidized to a hydrosoluble formazan dye by mitochondrial dehydrogenase in living cells. Briefly, purified RPMC or RBL-2H3 cells were washed once and plated into 96-well plates at a cell density of 2 × 10^5^ cells/mL (100 *μ*L/well). Next, cells were incubated in a humidified atmosphere of 95% air, 5% CO_2_, at 35°C or 37°C. After incubation for various time periods as indicated, the cell culture plates were centrifuged and the supernatant was discarded. Serum-free medium that contained CCK8 was added to each well (100 *μ*L/well) followed by a further incubation at 35°C or 37°C for 4 h. The absorbance was measured on an automated microplate reader (Bio-Rad, Japan) at 450 nm. This assay was repeated at least three times in sextuplicate.

### 2.6. Mast Cell Activation

RPMC and RBL-2H3 were activated by either IgE or c48/80 in 24-well plates (2.0 × 10^5^ cells/mL, 500 *μ*L/well). For IgE-mediated activation, mast cells were incubated with purified rat IgE (1 *μ*g/mL) for 1 h followed by two washes with PBS. Next, cells were incubated in serum-free medium containing 5 *μ*g/mL anti-rat IgE for 30 min. Alternatively, mast cells were incubated in serum-free medium containing 20 *μ*g/mL c48/80 for 30 min. In some experiments, cromolyn sodium (10 *μ*mol/L) was added 10 min before stimulation.

### 2.7. *β*-Hexosaminidase Release Measurement

*β*-Hexosaminidase release was assayed according to a previously published method [21]. Briefly, 50 *μ*L supernatant from cells that were appropriately activated was transferred to a 96-well plate and mixed with 50 *μ*L substrate solution followed by incubation for 2 h at 37°C. Glycine (0.2 M, pH 10) was added to each well to stop the reaction. The absorbance (OD) at 405 nm was measured with an automated microplate reader (Bio-Rad). To determine total release of mast cell granule-associated *β*-hexosaminidase (OD_total_), cells were treated with 0.1% (v/v) Triton X-100. The release rate was determined as follows: release (%) = (OD_eg_ − OD_bc_)/(OD_total_ − OD_bc_), where OD_eg_ and OD_bc_ represent the absorbance of the experimental group and blank control, respectively. The measurement was repeated at least three times in quadruplicate.

### 2.8. Measurement of Histamine Release

Histamine release was determined using a histamine ELISA kit from IBL International (Hamburg, Germany) [22]. After activation, cells were placed on ice for about 10 min followed by centrifugation at 500 g for 10 min. Supernatants were collected for measuring histamine content. The concentration of the fluorescent product formed during the reaction, which correlates to the histamine concentration, was measured using a fluorescence microplate reader set at *λ*_ex_ = 349 nm and *λ*_em_ = 448 nm. Total cellular release of histamine (*C*_total_) was achieved by treatment with 0.1% (v/v) Triton X-100. The release rate was determined as follows: release (%) = (*C*_eg_ − *C*_bc_)/(*C*_total_ − *C*_bc_), where *C*_eg_ and *C*_bc_ represent the concentrations of the experimental group and blank control, respectively. The measurement was repeated at least three times in quadruplicate.

### 2.9. Statistics

The results are expressed as means ± SE. Statistical evaluation of the data was performed by unpaired Student's *t*-test for comparisons between two groups and by ANOVA followed by Dunnett's test for comparisons between more than two groups. A value of *P* < 0.05 was regarded as significant.

## 3. Results

### 3.1. Culture Temperature Modulates Mast Cell Proliferation

**Figure 1:**
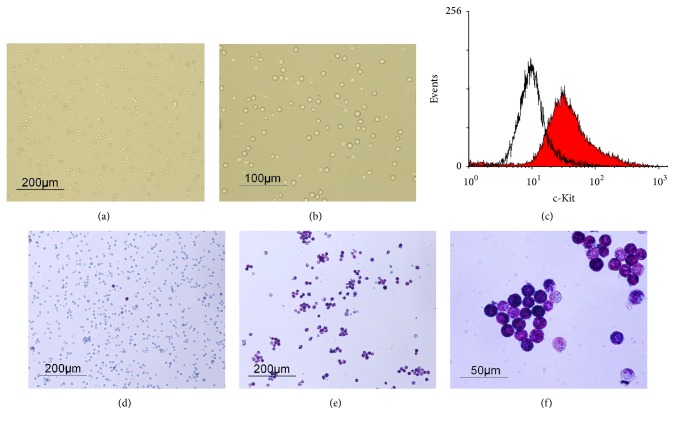
Purification of rat peritoneal mast cells. ((a) and (b)) Purified rat peritoneal mast cells were examined by phase-contrast microscopy at different magnification rates. (c) c-Kit expression by peritoneal cells before (line) and after (solid) purification was carried out by flow cytometric analysis. ((d)–(f)) Rat peritoneal cells either unpurified (d) or following purification ((e) and (f)) were stained with toluidine blue and examined by microscopy.

**Figure 2:**
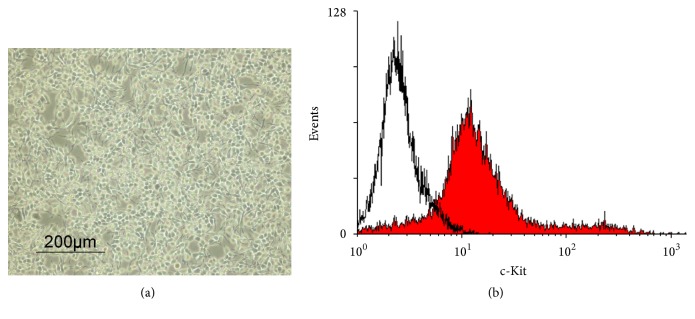
Purity analysis of RBL-2H3. (a) The morphology of RBL-2H3 cells was examined by phase-contrast photomicrography. (b) Cellular expression of c-Kit was analyzed by flow cytometric analysis. Line, isotype antibody control; solid, anti-c-Kit antibody.

RPMC were obtained and purified by Percoll density gradient centrifugation [23]. Following purification, mast cell morphology was examined by microscopy (Figures 1(a) and 1(b)). About 87% of the purified peritoneal mast cells expressed the mast cell marker c-Kit confirmed by flow cytometry, compared with the low frequency of c-Kit-expressing cells before purification (Figure 1(c)). The purity of RPMC reached greater than 90% by toluidine blue staining (Figures 1(e) and 1(f)) in contrast to the low frequency before purification (Figure 1(d)). Almost all the RBL-2H3 cells expressed c-Kit (Figure 2).

**Figure 3:**
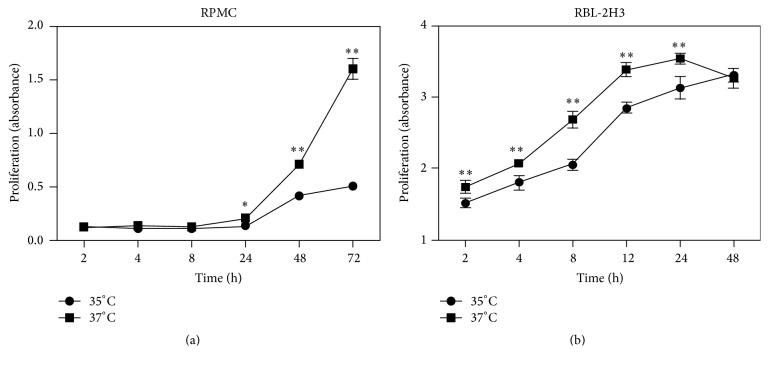
Growth curve of mast cells under different culture temperatures. Rat peritoneal mast cells (RPMC) (a) and RBL-2H3 (b) were cultured at 35°C or 37°C as indicated and cell proliferation was recorded. Data are expressed as the means ± SE (*n* = 6), representing at least three separate experiments. ^*⁎*^ *P* < 0.05; ^*⁎⁎*^ *P* < 0.01, compared between the two groups at the same time point.

Some of the RPMC and RBL-2H3 cells were cultured at 35°C instead of 37°C to simulate the lower temperature at body surface locations. RPMC did not show signs of proliferation until about 24 h after culture. After that, the proliferation of RPMC cultured at 35°C was significantly weaker than that at 37°C. Proliferation inhibition became more pronounced with an extended exposure time (Figure 3(a)). For RBL-2H3, growth inhibition as a result of low temperature culture was sustained from 2 h through 24 h (Figure 3(b)).

### 3.2. Degranulation of RPMC Is Modulated by Culture Temperature

**Figure 4:**
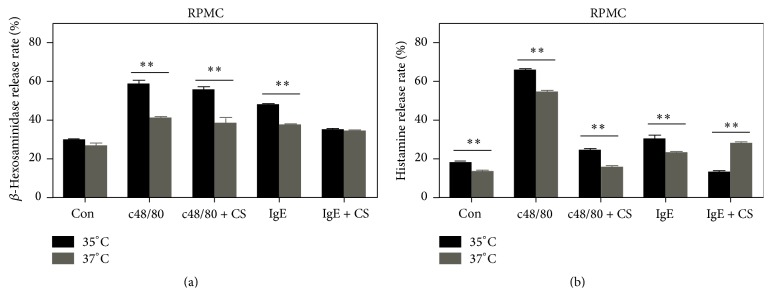
The effect of temperature on rat peritoneal mast cell degranulation. Rat peritoneal mast cells (RPMC) were left untreated (Con) or treated by either c48/80 or a combination of IgE and anti-IgE (IgE). In some experiments, cells were activated in the presence of the mast cell stabilizer cromolyn sodium (CS). *β*-Hexosaminidase (a) and histamine (b) release was measured. *β*-Hexosaminidase or histamine release is expressed as percent of the total (the means ± SE, *n* = 4, representing at least three separate experiments). ^*⁎⁎*^ *P* < 0.01.

Release of *β*-hexosaminidase and histamine is commonly used as readouts of mast cell degranulation. No significant difference in the basal release of *β*-hexosaminidase was observed when RPMC were cultured either at 35°C or at 37°C (Figure 4(a)). After treatment with c48/80 or IgE/anti-IgE, however, *β*-hexosaminidase release was more robust at 35°C compared to 37°C (Figure 4(a)). Histamine release profile was largely similar to that of *β*-hexosaminidase. For histamine release by RPMC, both spontaneous and stimulated release triggered by c48/80 or IgE cross-linking were greater at 35°C compared to 37°C (Figure 4(b)). The clinically widely used mast cell stabilizer, cromolyn sodium, exerted differential effects in our assay system. For *β*-hexosaminidase release, cromolyn failed to suppress c48/80-mediated degranulation either at 35°C or at 37°C. However, this inhibitor could partially inhibit IgE-mediated release of *β*-hexosaminidase at 35°C but not at 37°C (Figure 4(a)). For histamine release, cromolyn could substantially inhibit c48/80-mediated release at both temperatures. In contrast, cromolyn-mediated inhibition of degranulation triggered by IgE cross-linking was efficient at 35°C but not at 37°C (Figure 4(b)).

### 3.3. The Degranulation of RBL-2H3 Cells Is Modulated by Culture Temperature

**Figure 5:**
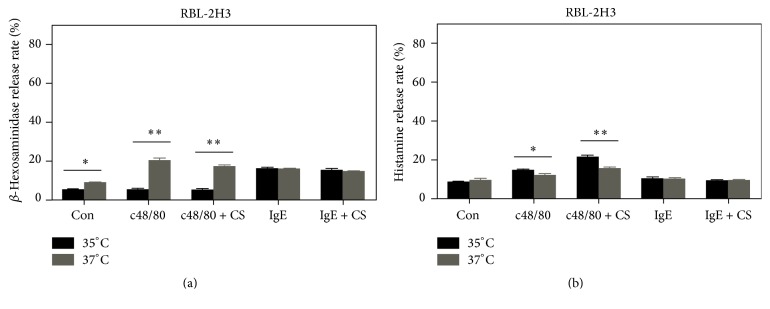
The effect of temperature on RBL-2H3 degranulation. RBL-2H3 cells were left untreated (Con) or treated by either c48/80 or a combination of IgE and anti-IgE. In some experiments, cells were activated in the presence of the mast cell stabilizer cromolyn sodium (CS). *β*-Hexosaminidase (a) and histamine (b) release was measured. *β*-Hexosaminidase or histamine release is expressed as percent of the total (the means ± SE, *n* = 4, representing at least three separate experiments). ^*⁎*^ *P* < 0.05; ^*⁎⁎*^ *P* < 0.01.

Both basal *β*-hexosaminidase release and the release induced by c48/80 at 37°C were remarkably higher than those at 35°C. In contrast, *β*-hexosaminidase release as a result of cross-linking of Fc*ε*RI by IgE reached similar levels at both 35°C and 37°C (Figure 5(a)). Slightly higher histamine release induced by c48/80 was observed at 35°C compared to 37°C (Figure 5(b)). IgE was not able to activate RBL-2H3 for pronounced histamine release (Figure 5(b)). Cromolyn failed to inhibit degranulation of RBL-2H3 cells (Figure 5).

## 4. Discussion

The effects of temperature on the functionality of mast cells have been previously explored [24–27], and the possible clinical implications in this regard have also been investigated [28]. However, these studies addressed drastically changed temperatures, for example, more than 10°C lower than the core body temperature of 37°C. In order to investigate the effect of a modestly lower temperature on mast cell functionality, we cultured RPMC and RBL-2H3 cells at 35°C and compared their performance with that of cells at 37°C.

Despite the fact that mast cell proliferation* in vitro* was slightly, but consistently, inhibited at 35°C over a broad range of time points, this may not necessarily affect* in vivo* mast cell physiology as terminally differentiated, tissue resident mast cells do not proliferate vigorously.

For RPMC degranulation triggered either by IgE receptor cross-linking or by c48/80, release of *β*-hexosaminidase and histamine was enhanced as a result of incubation at 35°C. Thus, we may speculate that peritoneal mast cells which are used to a core body temperature of 37°C could be more sensitive to external stimuli upon exposure to a lower temperature. In contrast, a lower incubation temperature differentially impacted RBL-2H3 cell line with regard to specific granule products. For histamine release, c48/80 induced a higher level of degranulation at 35°C compared with 37°C. However, the pattern was reversed for *β*-hexosaminidase release. This is possible as different granule products may be packaged in distinct granules which are not released upon a triggering stimulus at the same pace [29]. Cromolyn sodium is used as a stabilizer suppressing mast cell activation [30]. We did not observe its inhibitory effect on RBL-2H3 cells at all the conditions we tested. For RPMC activation, the effect of cromolyn sodium was also stimulus-specific and temperature-specific. The effectiveness of cromolyn sodium for topical application against skin allergies is reportedly controversial [31, 32]. Therefore, it would be interesting to investigate the property of cromolyn for inhibiting human mast cell degranulation at a lower temperature.

RBL-2H3 cells were originally isolated and cloned from Wistar rat basophilic leukemia cells [33]. These cells have high affinity IgE receptors, and they have been used extensively to study Fc*ε*RI-dependent activation pathways [34]. Here, we revealed certain differences between RBL-2H3 and* ex vivo* RPMC, as pointed out also by others [35].

## 5. Conclusions

In this study, we explored the effects of a modestly lower temperature on the proliferation and degranulation of RPMC as well as the rat mast cell line RBL-2H3, which may provide implications for both physiological and pathological functions of mast cells in certain tissues exposed to a lower temperature.
